# Wi-Fi/MARG Integration for Indoor Pedestrian Localization

**DOI:** 10.3390/s16122100

**Published:** 2016-12-10

**Authors:** Zengshan Tian, Yue Jin, Mu Zhou, Zipeng Wu, Ze Li

**Affiliations:** Chongqing Key Lab of Mobile Communications Technology, Chongqing University of Posts and Telecommunications, Chongqing 400065, China; tianzs@cqupt.edu.cn (Z.T.); zhoumu@cqupt.edu.cn (M.Z.); wuzipeng2014@gmail.com (Z.W.); lizecqupt@yahoo.com (Z.L.)

**Keywords:** indoor pedestrian localization, Wi-Fi, MARG, PDR, EKPF

## Abstract

With the wide deployment of Wi-Fi networks, Wi-Fi based indoor localization systems that are deployed without any special hardware have caught significant attention and have become a currently practical technology. At the same time, the Magnetic, Angular Rate, and Gravity (MARG) sensors installed in commercial mobile devices can achieve highly-accurate localization in short time. Based on this, we design a novel indoor localization system by using built-in MARG sensors and a Wi-Fi module. The innovative contributions of this paper include the enhanced Pedestrian Dead Reckoning (PDR) and Wi-Fi localization approaches, and an Extended Kalman Particle Filter (EKPF) based fusion algorithm. A new Wi-Fi/MARG indoor localization system, including an Android based mobile client, a Web page for remote control, and a location server, is developed for real-time indoor pedestrian localization. The extensive experimental results show that the proposed system is featured with better localization performance, with the average error 0.85 m, than the one achieved by using the Wi-Fi module or MARG sensors solely.

## 1. Introduction

The maturation and popularization of wireless communication technology has led to the emergence and development of various Location-Based Services (LBSs). LBSs are significantly required by the pedestrian in indoor environments, especially for emergency applications. In outdoor environments, the Global Position System (GPS) has been widely used and is able to provide high-enough localization accuracy. Unfortunately, due to the serious signal attenuation from the satellites, the localization performance in indoor environments becomes greatly degraded and even unavailable. Although various indoor localization systems, such as the Wi-Fi Received Signal Strength (RSS) [1,2], ZigBee [3], ultra-wideband [4], radio frequency identification [5], Bluetooth [6], and inertial sensors [7] based indoor localization systems, have been significantly studied, the design of the highly-accurate and low-cost indoor localization system still forms an interesting topic.

Wi-Fi RSS based indoor localization has been recognized as one of the most popular and representative indoor localization technologies. In the indoor Wi-Fi environment, commercial mobile devices can be used to collect the Wi-Fi RSS with the built-in Wi-Fi module. The signal propagation model based indoor localization approach generally requires the precise modelling of the RSS and physical distance, which is difficult to obtain [8]. The location fingerprint based indoor localization approach does not require foreknowing the locations of Access Points (APs), and thereby it adapts to the environmental well. This approach involves two phases: the offline and online phases. In the offline phase, the Wi-Fi RSS from APs is collected at each Reference Point (RP), and then a radio map is constructed based on the Wi-Fi RSS, MAC addresses, and location coordinates. Then, in the online phase, the new Wi-Fi RSS is collected in a real-time manner to be matched against the pre-constructed radio map for the localization. However, this method often leads to large localization errors and location jumps due to the irregular fluctuation of the RSS which is caused by the complex indoor environment. Besides, the computation cost involved in fingerprint matching generally increases with the increase of the dimensions of the target environment.

At the same time, with the rapid development of the Micro-Electro-Mechanical System (MEMS) technology, the Magnetic, Angular Rate, and Gravity (MARG) sensors have been embedded in various handheld devices such as the smartphones. By using the MARG sensors, the Pedestrian Dead Reckoning (PDR) approach, which relies on the previous locations, stride length, and walking direction to calculate the current location of the pedestrian, has become very popular for pedestrian localization. Some previous studies use the empirical models about the walking state to estimate the stride length [9], meanwhile the existing heading estimation approaches [10,11,12,13] usually rely on the gyroscope solely or the fusion of the gyroscope and magnetometer to calculate the user heading. However, the stride length cannot be accurately estimated in each step when the pedestrian walks in changing scenarios and the heading calculation may also not be accurate when the measurement data suffer from magnetic interference and gyroscope drift. Although the PDR approach is able to achieve high localization accuracy in short time, the drift of the estimated locations will occur as the time goes on, since the inertial measurement unit easily suffers from the accumulative errors of the estimated stride length and walking direction. On the contrary, the Wi-Fi RSS based localization approach estimates the absolute locations of the pedestrian, which can help to mitigate the accumulative errors of the PDR. These two localization approaches are complementary to each other for the sake of improving the indoor localization accuracy.

Although a large number of fusion algorithms have been studied for integrating the PDR and Wi-Fi localization, many critical issues are still open. The current fusion algorithms mainly focus on deploying the Particle Filter (PF) [14,15], Kalman Filter (KF) [16], and the improved algorithm based on the previous two algorithms. The PF helps a lot in improving the localization accuracy, especially for the nonlinear and non-Gaussian systems, but it involves high computation cost. In addition, it is difficult to obtain the optimal Importance Density Function (IDF) which is used for generating the high-quality particles. The KF is based on the assumption that the noise obeys the Gaussian distribution with zero mean, which cannot be easily satisfied in the actual indoor environment.

**Figure 1:**
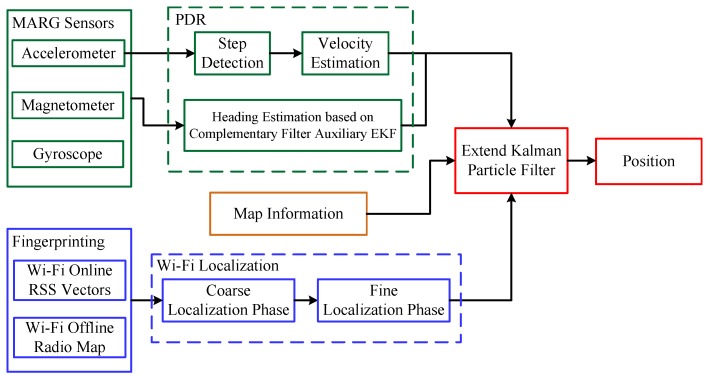
Architecture of the proposed system.

In summary, the most important contribution of this paper is to design a novel indoor localization system by using built-in MARG sensors and Wi-Fi module. To improve the robustness and effectiveness of our system, we accomplish the design and optimization of three main modules, including the enhanced Wi-Fi localization, enhanced Pedestrian Dead Reckoning, and Extended Kalman Particle Filter (EKPF) based fusion algorithms. We propose integrating the map information and the results of the PDR and Wi-Fi localization by using the EKPF, which depends on the EKF to optimize the IDF with the purpose of reducing the number of particles and approximating the true posterior distribution. In the PDR, by considering magnetic interference and gyroscope drift, the complementary filter auxiliary Extended Kalman Filter (EKF) is used to integrate the tri-axial accelerometer, tri-axial gyroscope, and tri-axial magnetometer for the sake of estimating the heading of the pedestrian. In addition, we adopt a neural network to estimate the parameters of the stride length model as well as calculating the velocity per each second. For the Wi-Fi localization, we apply the improved affinity propagation clustering to save the computation time of fingerprint matching and reduce the number of large localization errors. This process is shown in Figure 1 and the detailed steps will be discussed in the following sections.

The rest of this paper is organized as follows. Section 2 introduces the related works. Section 3 describes the proposed system including the PDR and Wi-Fi localization approaches, as well as the related fusion algorithm. In Section 4, the extensive experiments are carried out in an actual indoor environment. Finally, Section 5 concludes the paper and provides some future directions.

## 2. Related Works

In recent decades, various low-cost inertial sensors have been equipped in the off-the-shelf smartphones due to many functional requirements, and meanwhile the Wi-Fi network has also been widely deployed in the indoor environment. Thus, the PDR and Wi-Fi localization approaches are recognized as the two most promising approaches used for indoor localization. The earliest work focusing on the fusion of the PDR and Wi-Fi localization is discussed in [17]. The authors achieve the integration of the inertial sensors and Wi-Fi module by using both the PF and KF. In concrete terms, as one of the most important parameters in the PDR, the heading of the pedestrian is estimated by the KF, which fuses the output of the gyroscope and the angle of trajectory calculated by the Wi-Fi localization. However, the approach is limited in terms of localization accuracy since their performance significantly depends on the robustness of Wi-Fi localization. In [18], the authors propose an adaptive integration frame to fuse localization information from the GPS/MARG and Wi-Fi/MARG integrated subsystems, and meanwhile use a Sequential Importance Resampling (SIR) PF approach to integrate the Wi-Fi RSS measurements and data from the MARG sensors. The particles are calculated by using the velocity and attitude coming from MARG sensors, while ignoring the impact of Wi-Fi observation data on particle sampling. In this paper, we depend on the EKF with observation data from the Wi-Fi and MARG sensors to optimize the IDF for the sake of reducing the number of particles and approximating the true posterior distribution.

The work studied in [19] focuses on integrating the gyroscope, accelerometer, and Wi-Fi fingerprint by using a complementary EKF. This approach estimates the heading of the pedestrian, based on the data from the gyroscope and the linear model between the step length and step frequency. An auto-calibration process is introduced in [20] to reduce the measurement error of heading direction as well as the estimation error of step length during movement.

Considering the variation of Wi-Fi RSS, computation cost involved in fingerprint matching, and utilization of the low-cost inertial sensors built-in smartphones, the refresh rate model of the Wi-Fi signal is introduced in [21] to optimize the Wi-Fi localization, and meanwhile the affinity propagation clustering is discussed in [22] to reduce the computation cost for the positioning. At the same time, an empirical model constructed from the individual height and peak-to-peak magnitude of the acceleration in each step is developed in [23] to estimate the stride length of the pedestrian. The quaternion based heading estimation approach [24] is capable of compensating the drift of heading estimation with low computation cost. Different from the approaches above, in this paper, we first rely on a new velocity calculation algorithm to reduce the error of speed estimation, and meanwhile integrate the tri-axial gyroscope, tri-axial accelerometer, and tri-axial magnetometer by using the complementary filter auxiliary EKF [25] to improve the accuracy of heading estimation. Second, we apply the improved affinity propagation clustering to reduce the number of large localization errors, as well as achieving the low computation complexity. Finally, the extend Kalman particle filter is applied to accomplish the Wi-Fi/MARG fusion, which is capable of eliminating the location jumps caused by the Wi-Fi localization and the accumulative errors of the PDR.

## 3. System Description

### 3.1. Pedestrian Dead Reckoning

In the PDR, the previous location, estimated stride length, and heading are used to reckon the current location of the pedestrian. To reduce the accumulative error, we propose an enhanced algorithm for the estimation of the velocity and walking direction.

#### 3.1.1. Velocity Estimation

**Figure 2:**
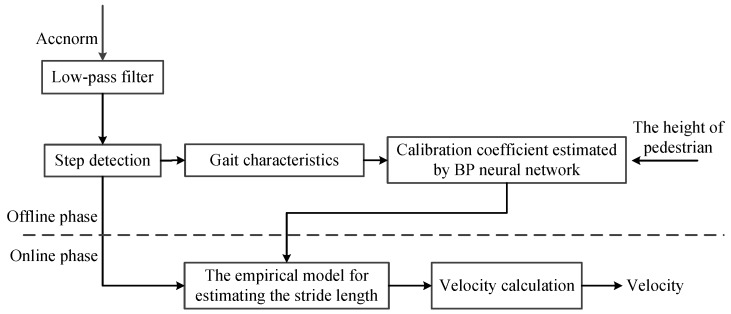
Block diagram of velocity estimation.

We rely on the data from the tri-axial accelerometer to estimate the velocity of the pedestrian, as shown in Figure 2. In the offline phase, we optimize the calibration coefficient of an empirical model by using the Back Propagation (BP) neural network [26], and then use this model to estimate the stride length. In concrete terms, we first calculate the relative magnitude of acceleration, which is defined as the difference between the local and measuring acceleration, namely Accnorm, and meanwhile use a low-pass filter to eliminate the noise involved in Accnorm. Second, we perform the step detection to extract the gait characteristics, such as the gait frequency and statistical quantities of acceleration magnitude, i.e., mean, variance, maximum, and minimum of Accnorm. Finally, we optimize the calibration coefficient based on the BP neural network. In the online phase, we estimate the stride length and velocity of the pedestrian by using the empirical model and step detection respectively in a real-time manner.

● Step Detection

**Figure 3:**
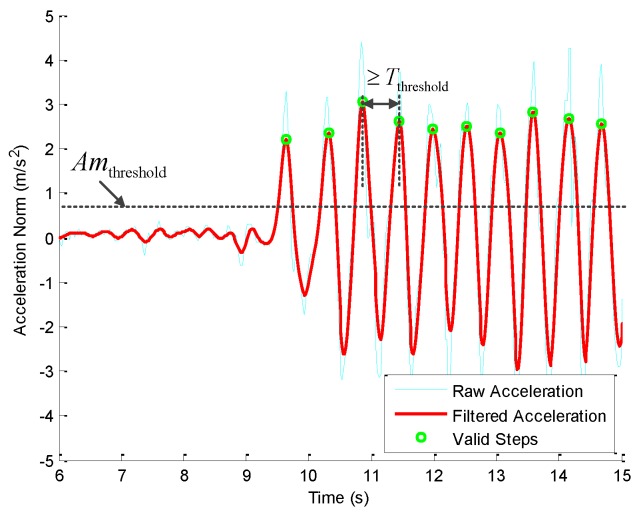
Results of step detection.

Due to the error of the installation of inertial instruments, we rely on the periodic variation of Accnorm to detect the gait of the pedestrian. In Figure 3, the abnormal peaks are caused by the random self-dithering of the human body and the noise of sensors. By setting the interval between every two adjacent local maximum values as no smaller than the time threshold $T_{\mathrm{threshold}}$ and the local maximum values as larger than the amplitude threshold $Am_{\mathrm{threshold}}$, the valid steps (with green circles) are detected in Figure 3. Considering that the pedestrian approximately takes two steps for each second and the updating rate of the data from the MARG sensors is 50 Hz, we set $T_{\mathrm{threshold}}=0.3$ s and $Am_{\mathrm{threshold}}=0.8$ m/s${}^{2}$.

● Velocity Calculation

The velocity estimation varies from step to step, especially when the stride length is treated as a constant. We use the empirical model in Equation (1) to estimate the stride length, $L_{k}$, based on the acceleration measurement.

(1) $$L_{k}=\rho \sqrt[{4}]{acc_{\max}^{k}-acc_{\min}^{k}}$$

where $acc_{\max}^{k}$ and $acc_{\min}^{k}$ are the maximum and minimum values of Accnorm for the *k*-th step. *ρ* is the calibration coefficient which equals the ratio between the real distance $d_{\mathrm{real}}$ and estimated distance $d_{\mathrm{estimated}}$. According to the biomechanical property, the stride length is determined by the step frequency and height of the pedestrian. The nonlinear model for the calibration coefficient *ρ* is constructed from the step frequency, *f*, height of the pedestrian, *h*, and mean and variance of accelerometer magnitude, Amean and Avar, as shown in Equation (2). In our system, the coefficient *ρ* is estimated by using the BP neural network.

(2) ρ=Φ(f,Amean,Avar,h)

**Figure 4:**
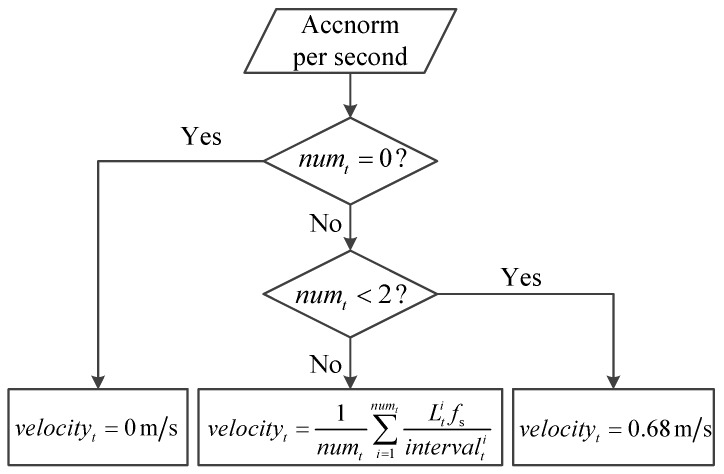
Block diagram of velocity calculation.

Figure 4 shows the block diagram of velocity calculation. By performing the step detection, we can obtain the number of steps, $num_{t}$, in each second. In the *t*-th second, if $num_{t}=0$, the velocity is set as 0 m/s, which indicates that the pedestrian is not moving, if $0<num_{t}<2$, the velocity is set as 0.68 m/s, which indicates that the pedestrian walks for one step, and otherwise, we calculate the velocity by

(3) $$velocity_{t}=\frac{1}{num_{t}}\sum_{i=1}^{num_{t}}\frac{L_{t}^{i}f_{s}}{interval_{t}^{i}}$$

where $L_{t}^{i}$ is the *i*-th stride length, which is calculated by Equation (1). $interval_{t}^{i}$ is the number of sampling points between every two valid local maximum values. $f_{s}=50\;\;\mathrm{Hz}$ is the updating rate of the data from the MARG sensors.

#### 3.1.2. Heading Estimation

**Figure 5:**
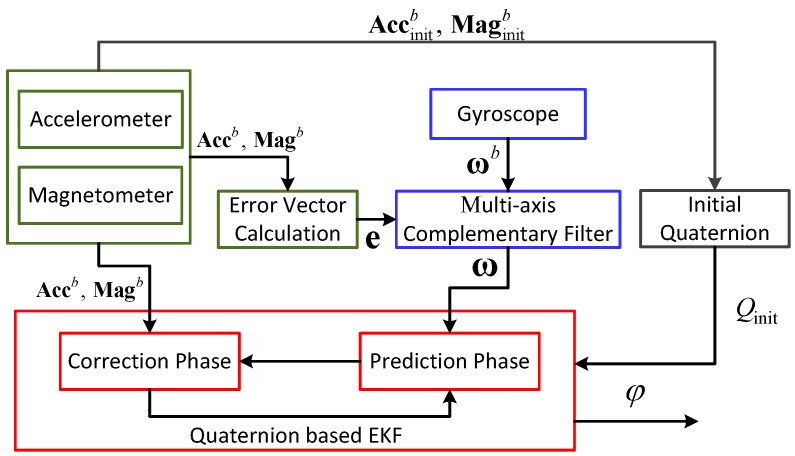
Block diagram of heading estimation.

The state-of-the-art smartphones are generally equipped with the MARG sensors, like the tri-axial accelerometer, magnetometer, and gyroscope. Theoretically, the attitude of the carrier can be estimated by using the data in the gravity and geomagnetic fields, or the data from the gyroscope. However, the accelerometer and magnetometer are easily interfered with by the noise of sensors; meanwhile, the angular rate obtained from the gyroscope always drifts for a long time. To solve these problems, we adopt the complementary filter auxiliary EKF to estimate the heading of the pedestrian, as shown in Figure 5.

● Initial quaternion calculation

Based on the Euler theorem of the rigid body rotation [27], we use the quaternion matrix to describe the rotation of the body frame relative to the East-North-Up (ENU) reference frame, as shown in Equation (4).

(4) $$H_{n}^{b}\left(q\right)=\left[\begin{array}{c} \begin{array}{ccc} q_{1}^{2}+q_{2}^{2}-q_{3}^{2}-q_{4}^{2} & 2(q_{2}q_{3}+q_{1}q_{4}) & 2(q_{2}q_{4}-q_{1}q_{3}) \end{array}\\ \begin{array}{ccc} 2(q_{2}q_{3}-q_{1}q_{4}) & q_{1}^{2}-q_{2}^{2}+q_{3}^{2}-q_{4}^{2} & 2(q_{3}q_{4}+q_{1}q_{2}) \end{array}\\ \begin{array}{ccc} 2(q_{2}q_{4}+q_{1}q_{3}) & 2(q_{3}q_{4}-q_{1}q_{2}) & q_{1}^{2}-q_{2}^{2}-q_{3}^{2}+q_{4}^{2} \end{array} \end{array}\right]$$

where $H_{n}^{b}\left(q\right)$ is the attitude matrix. $q={\left[\begin{array}{cccc} q_{1} & q_{2} & q_{3} & q_{3} \end{array}\right]}^{T}$ is the attitude quaternion, which is set as a unit vector. We calculate the initial attitude quaternion $Q_{\mathrm{init}}$ by using the data from the accelerometer and magnetometer, as shown in Equation (5).

(5) $$\left\{\begin{array}{c} {\mathrm{Acc}}_{\mathrm{init}}^{b}=H_{n}^{b}\left(q\right)\cdot {\left[\begin{array}{ccc} 0 & 0 & g \end{array}\right]}^{T}\\ {\mathrm{Mag}}_{\mathrm{init}}^{b}=H_{n}^{b}\left(q\right)\cdot {\left[\begin{array}{ccc} 0 & b_{y}^{n} & b_{z}^{n} \end{array}\right]}^{T} \end{array}\right.$$

where the initial measurement vectors from the tri-axial accelerometer and magnetometer are ${\mathrm{Acc}}_{\mathrm{init}}^{b}={\left[\begin{array}{ccc} a_{x}^{b} & a_{y}^{b} & a_{z}^{b} \end{array}\right]}^{T}$ and ${\mathrm{Mag}}_{\mathrm{init}}^{b}={\left[\begin{array}{ccc} m_{x}^{b} & m_{y}^{b} & m_{z}^{b} \end{array}\right]}^{T}$ respectively. *g* is the local gravity acceleration. $b_{y}^{n}$ and $b_{z}^{n}$ are the northward and perpendicular components of the local earth magnetic field in the ENU reference frame respectively. Finally, by using the method in [28] to calculate the magnetic heading angle, the initial heading of the pedestrian is calculated by

(6) $$\varphi_{\mathrm{init}}=\arctan\left(m_{x}^{b},m_{y}^{b}\right)$$

● Gyroscope error modification

The accumulative error of angular rate which is caused by the drift of the gyroscope significantly deteriorates the precision of heading estimation. Although the attitude angular, which is calculated by using the data from the accelerometer and magnetometer, is not featured with high precision, it is able to remain stable for a long time. The gyroscope, accelerometer, and magnetometer can be used to complement each other since the gyroscope exhibits the high-pass property, while the accelerometer and magnetometer exhibit the low-pass property. Therefore, the complementary filter method, which relies on the complementary characteristics of the MARG sensors to modify the data from the gyroscope, improves the precision of attitude estimation. The angular velocity $\theta \left(k\right)$ in time domain is modified as

(7) $$\dot{\theta }\left(k\right)=K\left(\theta_{\mathrm{ref}}\left(k\right)-\theta \left(k\right)\right)+\omega^{b}\left(k\right)$$

where $\omega^{b}\left(k\right)$ is a vector of the *k*-th measured angular rate. $\theta_{\mathrm{ref}}\left(k\right)$ is the *k*-th attitude angular which is calculated by using the data from the magnetometer. *K* is an adjustable parameter. However, the modified attitude angular cannot be easily obtained from Equation (7). To solve this problem, we select the Mahony based multi-axis complementary filter which is also used in [29] to modify the angular rate obtained from the gyroscope.

For pedestrians with a low walking speed, the attitude angular always has small variation in every 0.02 s. Thus, we use the estimated attitude angular in the latest updating to construct the attitude matrix for the sake of modifying the drift of the gyroscope. Specifically, we standardize the newly collected measurement vectors in the acceleration and geomagnetic field, $\mathrm{Ac}c^{b}$ and $\mathrm{Ma}g^{b}$, in the body coordinate frame, and then estimate the projection vectors in the acceleration and geomagnetic field, $a^{s}$ and $m^{s}$, by using the quaternion rotation matrix. Since the measurement and projection vectors are in the same coordinate frame, the cross product of them can be described as

(8) $$e_{a}=a^{s}\times \mathrm{Ac}c^{b}$$

(9) $$e_{m}=m^{s}\times \mathrm{Ma}g^{b}$$

We select the error vector e as the input of the Proportion Integral (PI) controller [30] to calculate the modification of angler rate δω, such that

(10) $$\delta \omega =Kp\cdot e\left(k\right)+Ki\sum_{j=0}^{k}\left(e\left(j\right)\cdot \Delta t\right)$$

where Kp is the proportional integral coefficient. Ki is the control integral coefficient. Δt is the integral time constant. $e=e_{a}+e_{m}$ is the error in total.

After the value δω is obtained, the modified angular rate equals to

(11) $$\omega \left(k\right)=\delta \omega +\omega^{b}\left(k\right)$$

where $\omega \left(k\right)$ is the *k*-th modified angular rate vector.

● Attitude angular estimation

Since the data from the gyroscope can be used to estimate the attitude angular based on the differential equation of the rigid body motion, we use the EKF to estimate the attitude angular. In addition, we rely on the measurement vectors in the gravity and geomagnetic fields to modify the attitude angular.

By setting the state variable as a quaternion vector, Q, and measurement variables as the measurement vectors in the gravity and geomagnetic fields, the state and measurement equations can be constructed as

(12) $$\left\{\begin{array}{c} Q\left(k\right)=\left(I+\frac{1}{2}\Lambda \left(\omega \left(k\right)T_{S}\right)\right)Q\left(k-1\right)+\varpi \left(k\right)\\ \left[\begin{array}{c} \mathrm{Ac}c^{b}\left(k\right)\\ \mathrm{Ma}g^{b}\left(k\right) \end{array}\right]=\left[\begin{array}{c} \begin{array}{cc} H_{n}^{b}\left(Q\left(k\right)\right) & 0 \end{array}\\ \begin{array}{cc} 0 & H_{n}^{b}\left(Q\left(k\right)\right) \end{array} \end{array}\right]\left[\begin{array}{c} G\\ b \end{array}\right]+\nu \left(k\right) \end{array}\right.$$

where $G={\left[\begin{array}{ccc} 0 & 0 & g \end{array}\right]}^{T}$ is the local gravity acceleration vector. $b={\left[\begin{array}{ccc} 0 & b_{y}^{n} & b_{z}^{n} \end{array}\right]}^{T}$ is the local earth magnetic field vector in the ENU reference frame. I is the identity matrix. $T_{S}$ is the system interval. $\omega \left(k\right)=\left[\begin{array}{ccc} \omega_{x}^{b}\left(k\right) & \omega_{y}^{b}\left(k\right) & \omega_{z}^{b}\left(k\right) \end{array}\right]$ is the modified angular rate in the $kT_{S}$-th time interval. $\varpi \left(k\right)$ is the matrix of processing noise and $\nu \left(k\right)$ is the matrix of observation noise. $\Lambda \left(\omega \left(k\right)T_{S}\right)$ is calculated by

(13) $$\Lambda \left(\omega \left(k\right)T_{S}\right)=\left[\begin{array}{cccc} 0 & -\omega_{x}^{b}T_{S} & -\omega_{y}^{b}T_{S} & -\omega_{z}^{b}T_{S}\\ \omega_{x}^{b}T_{S} & 0 & \omega_{z}^{b}T_{S} & -\omega_{y}^{b}T_{S}\\ \omega_{y}^{b}T_{S} & -\omega_{z}^{b}T_{S} & 0 & \omega_{x}^{b}T_{S}\\ \omega_{z}^{b}T_{S} & \omega_{y}^{b}T_{S} & -\omega_{x}^{b}T_{S} & 0 \end{array}\right]$$

Finally, based on the recursive filtering process in EKF by Equation (12), including the correction phase and prediction phase, we can obtain the optimal quaternion vector in each second, and then use the newly updated quaternion to estimate the heading of the pedestrian by Equation (14).

(14) $$\varphi =\arctan(-\frac{2(q_{1}q_{2}+q_{0}q_{3})}{q_{0}^{2}+q_{1}^{2}-q_{2}^{2}-q_{3}^{2}})$$

### 3.2. Wi-Fi Localization

**Figure 6:**
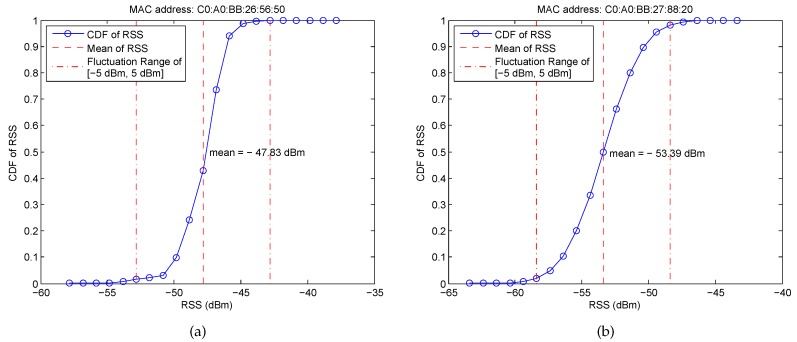
CDFs of RSS from two different APs for 3 min. (**a**) RSS from AP with the MAC address C0: A0: BB: 26: 56: 50; (**b**) RSS from AP with the MAC address C0: A0: BB: 27: 88: 20.

**Figure 7:**
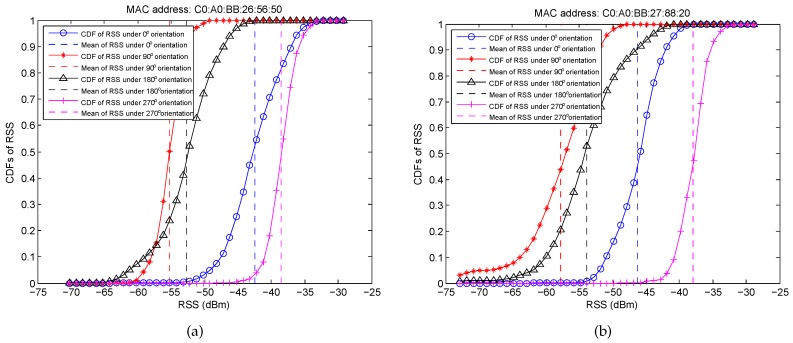
CDFs of RSS under four different orientations for 3 min. (**a**) RSS from AP with the MAC address C0: A0: BB: 26: 56: 50; (**b**) RSS from AP with the MAC address C0: A0: BB: 27: 88: 20.

The Wi-Fi RSS is the superposition value of multiple signal paths, which easily suffers from the multipath interference. Figure 6 shows an example of the Cumulative Distribution Functions (CDFs) of RSS from two different APs at a given RP for 3 min. It can be obtained that the RSS is not stable, even in a short time duration, and the probability of RSS fluctuation around its mean within the range of [−5 dBm, 5 dBm] is higher than 90%. Furthermore, to examine the impact of the blocking of human body on RSS fluctuation, we design another experiment in which the RSS is collected under four different orientations, i.e., $0^{\circ }$, $90^{\circ }$, $180^{\circ }$, $270^{\circ }$, as shown in Figure 7. From these figures, we can find that (i) RSS fluctuation becomes significantly large and even reaches more than 20 dBm by the blocking of the human body; (ii) different orientations result in a different mean of RSS and different ranges of RSS fluctuation; and (iii) RSS from different APs at each RP generally has a different mean of RSS and different ranges of RSS fluctuation, which guarantees the effectiveness of Wi-Fi localization.

There are two phases involved in Wi-Fi localization, namely the offline phase and online phase. In the offline phase, we collect the RSS sequences labelled by timestamps to construct a radio map, in which the orientation of human body, MAC address of each AP, and location of each RP are also recorded. Then, based on the similarities of the RSS and the corresponding geographic location coordinates, affinity propagation clustering is performed to obtain a batch of clusters of RPs.

In the online phase, the orientations of human body are estimated by using the heading estimation approach, which has been carefully discussed in Section 3.1. Then, the newly collected RSS and the related estimated orientation are matched against the pre-constructed radio map to obtain the best-matching cluster of RPs. After that, the locations of the pedestrian are estimated by performing the WKNN on the selected RPs in the best-matching cluster.

#### 3.2.1. Offline Phase

● Radio map construction

To deal with the problem that the RSS collected under different orientations of the human body may be featured with significant variation, we construct eight radio maps, including the four under the orientations $0^{\circ }$, $90^{\circ }$, $180^{\circ }$, $270^{\circ }$, and the other four constructed by using the mean of two radio maps with the neighbouring orientations. By assuming that there are *M* APs and *P* RPs, the RSS vector is notated as

(15) $$\psi_{i}^{k}={\left[\psi_{i,1}^{k},\cdots ,\psi_{i,j}^{k},\cdots ,\psi_{i,M}^{k}\right]}^{T}$$

where $\psi_{i,M}^{k}\;\;\;\left(1\leq i\leq P,\begin{array}{c} 1\leq j\leq M \end{array},\begin{array}{c} 1\leq k\leq 8 \end{array}\right)$ is the mean of RSS from the *j*-th AP, at the *i*-th RP, and under the *k*-th orientation. The radio map with respect to the *k*-th orientation, $\Lambda^{k}$, is constructed as

(16) $$\Lambda^{k}={\left[\begin{array}{c} \begin{array}{cccc} \psi_{1}^{k} & \psi_{2}^{k} & \cdots & \psi_{P}^{k} \end{array}\\ \begin{array}{cccc} x_{1}^{} & \;\;x_{2}^{} & \;\;\;\;\cdots & x_{P}^{} \end{array}\\ \begin{array}{cccc} y_{1}^{} & \;\;y_{2}^{} & \;\;\;\cdots & y_{P}^{} \end{array} \end{array}\right]}^{T}$$

● Affinity propagation clustering

For simplicity, we assume that the RSS vectors collected at the RPs *i* and *j* are $\psi_{i}$ and $\psi_{j}$ respectively. To describe the similarity of every two RPs, we set the similarity function as

(17) $$s\left(i,j\right)=-{∥\psi_{i}-\psi_{j}∥}_{2},\forall i,j\neq i\in \left\{1,2,\cdots ,P\right\}$$

During affinity propagation clustering, we define two types of messages which are transmitted among different RPs, namely the responsibility message and availability message. The responsibility message, r(i,j), describes the credibility about how suitably the RP *j* serves as the clustering centre for the RP *i*, while the availability message, a(i,j), describes the credibility about how suitably the RP *i* selects the RP *j* as its corresponding clustering centre.

In general, the larger sum of the values r(i,j) and a(i,j) indicates the higher likelihood of the RP *j* to be selected as the clustering centre. In addition, the responsibility and availability messages are updated iteratively among different RPs until the optimal clustering centres and the corresponding clusters have been obtained. We define $H^{k}$ as the set of clustering centres and $C_{j}^{k}$ as the set of RPs in the *j*-th cluster for the radio map with the *k*-th orientation. Considering that some clusters of RPs may contain the singular points, which are geographically far away from the clustering centres of the corresponding clusters, we use the outlier detection method [31] to eliminate the singular points after obtaining the set of clusters by the APC.

#### 3.2.2. Online Phase

The online phase contains two main steps, namely the coarse localization step and fine localization step. The newly collected RSS vector is notated as

(18) $$\psi_{A}={\left[\psi_{A,1},\psi_{A,2},\cdots ,\psi_{A,M}\right]}^{T}$$

**Figure 8:**
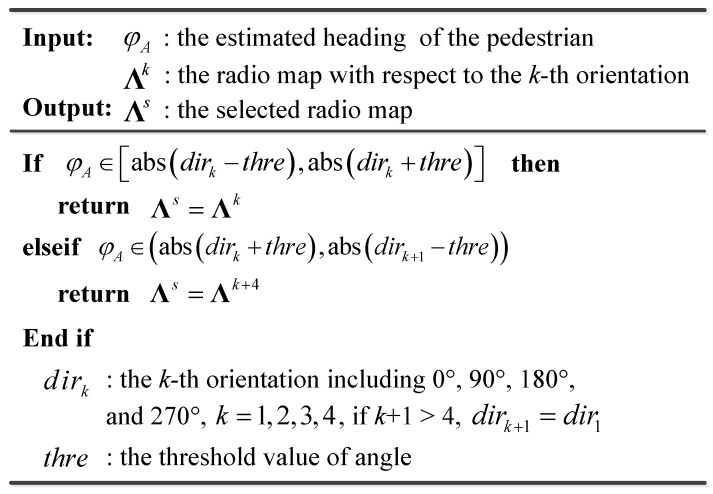
Pseudo code of the selection of the best-matching radio map.

In the coarse localization step, we rely on the estimated heading of the pedestrian, $\varphi_{A}$, to obtain the best-matching radio map. The pseudo code of this process is shown in Figure 8. Then, we match the newly collected RSS vector $\psi_{A}$ against the selected radio map to obtain the best-matching cluster of RPs for the fine localization.

The clustering centre of the best-matching cluster is defined as

(19) $$j^{\prime }=\arg\min_{j\in H^{s}}{∥\psi_{A}-\psi_{j}^{s}∥}^{2}$$

where $H^{s}$ is the set of clustering centres for the selected radio map $\Lambda^{s}$. $\psi_{j}^{s}$ is the RSS vector collected at the *j*-th RP and in the radio map with the *s*-th orientation.

In the fine localization step, we perform the WKNN on some specific RPs in the best-matching cluster to locate the pedestrian. Specifically, we calculate the Euclidean distance between the newly collected RSS and the RSS at each RP in the best-matching cluster by

(20) $$d_{j}=\sqrt{\sum_{i=1}^{M}{\left(S_{ij}-\psi_{A,i}\right)}^{2}}$$

where $S_{ij}\left(i=1,2,\cdots ,M,\;j=1,2,\cdots ,n\right)$ is the RSS collected from the *i*-th AP, at the *j*-th RP, and in the best-matching cluster. *n* is the number of RPs in the cluster $C_{j^{\prime }}^{s}$. $\psi_{A,i}$ is the newly collected RSS from the *i*-th AP. The estimated location of the pedestrian, $\tilde{L}=\left(\tilde{x},\tilde{y}\right)$, is as follows.

(21) $$\tilde{L}=\sum_{i=1}^{K}\omega_{i}L_{i}$$

(22) ωi=1di∑m=1K1dm2

where $L_{i}=\left(x_{i},y_{i}\right)\begin{array}{c} \left(i=1,2,\cdots ,K\right) \end{array}$ is the *i*-th selected RP with the smallest Euclidean distance in the best-matching cluster. *K* is the number of selected RPs used for the WKNN. $\omega_{i}$ is the weight of the *i*-th selected RP.

### 3.3. Extended Kalman Particle Filter

The performance of the conventional particle filter degrades significantly when there is a large difference between the optimal IDF and real posterior distribution function [32]. To solve this problem, we use the EKF to obtain the optimal IDF with the purpose of guaranteeing that the particles are located in the high likelihood region. By fusing the Wi-Fi fingerprint information and the data from the MARG sensors, the proposed EKPF is able to reduce the number of particles with low computation cost, as well as improving the localization accuracy.

For the PDR, we construct the state equation in Equation (23); meanwhile, by assuming that the state variable is stable in a short time, we also construct the observation equation in Equation (24).

(23) $$X_{k}=\left[\begin{array}{c} Eloc_{k}\\ Nloc_{k}\\ vel_{k}\\ head_{k} \end{array}\right]=\left[\begin{array}{c} Eloc_{k-1}+vel_{k}\cdot \sin\left(head_{k}\right)\\ Nloc_{k-1}+vel_{k}\cdot \cos\left(head_{k}\right)\\ vel_{k-1}\\ head_{k-1} \end{array}\right]+W_{k}$$

where $X_{k}$ is the state variable vector. $Eloc_{k}$ and $Nloc_{k}$ are the optimal eastern and northern locations. $vel_{k}$ and $head_{k}$ are the optimal velocity and heading of the pedestrian. $W_{k}$ is the matrix of processing noise which obeys the Gaussian distribution with the zero mean and covariance matrix $Q_{w}$.

(24) $$Z_{k}=H\cdot X_{k}+V_{k}$$

where $Z_{k}={\left[\begin{array}{cccc} Eloc_{k}^{WiFi} & Nloc_{k}^{WiFi} & vel_{k}^{MARG} & head_{k}^{MARG} \end{array}\right]}^{T}$ is the measurement variable vector. $Eloc_{k}^{WiFi}$ and $Nloc_{k}^{WiFi}$ are the current eastern and northern locations estimated by Wi-Fi localization. $vel_{k}^{MARG}$ and $head_{k}^{MARG}$ are the current velocity and heading of the pedestrian estimated by using the PDR. $V_{k}$ is the matrix of observation noise which obeys the Gaussian distribution with the zero mean and covariance matrix $R_{v}$.

The steps of the proposed EKPF are summarized as follows.

First of all, due to the fact that the PDR is a relative localization approach, we rely on the Wi-Fi localization to estimate the absolute initial location of the pedestrian, $\left(Eloc_{0},Nloc_{0}\right)$. At the same time, the initial particles $\left\{\left.X_{0}^{i},i\in 1,\cdots ,M\right\}\right.$, where *M* is the number of particles, satisfy

(25) $$X_{0}^{i}∼p\left(X_{0}\right)=N\left(X_{0},Q_{0}\right)$$

where $X_{0}={\left[\begin{array}{cccc} Eloc_{0} & Nloc_{0} & 0 & 0 \end{array}\right]}^{T}$ is the mean of the initial particles. $p\left(X_{0}\right)$ is the proposal distribution function which obeys the Gaussian distribution. $Q_{0}$ is the covariance matrix of the initial processing noise which is used to describe the dispersion degree of the initial particles. The weight of the *i*-th initial particle is set as ω0i=11MM.

Second, we apply the EKF [33] to update the state of particles in Equations (26)–(30), and meanwhile fuse the measurement information to optimize the weights of particles in Equation (31)–(32).

(26) $${\hat{X}}_{\left.k\right|k-1}^{i}=f\left({\tilde{X}}_{k-1}^{i}\right)$$

(27) $$P_{\left.k\right|k-1}^{i}=F_{k,i}P_{k-1,i}F_{k,i}^{T}+Q_{w}^{k}$$

(28) $$K_{k}^{i}=P_{\left.k\right|k-1}^{i}H_{k,i}^{T}{\left[H_{k,i}^{}P_{\left.k\right|k-1}^{i}H_{k,i}^{T}+R_{v}^{k}\right]}^{-1}$$

(29) $${\tilde{X}}_{k}^{i}={\hat{X}}_{\left.k\right|k-1}^{i}+K_{k}^{i}\left[Z_{k}-H\left({\hat{X}}_{\left.k\right|k-1}^{i}\right)\right]$$

(30) $$P_{k,i}=P_{\left.k\right|k-1}^{i}-K_{k}^{i}H_{k,i}^{}P_{\left.k\right|k-1}^{i}$$

(31) $$\begin{array}{c} q_{k}^{i}∼\pi_{N}\left(X_{k}\left|Z_{1:k}\right.\right)\\ \begin{array}{cc} & = \end{array}\frac{1}{{\left(2\pi \right)}^{m/2}{\left|R_{v}^{k}\right|}^{1/2}}\exp\left(\frac{-{\left[Z_{k}-H\left({\hat{X}}_{\left.k\right|k-1}^{i}\right)\right]}^{T}{\left(R_{v}^{k}\right)}^{-1}\left[Z_{k}-H\left({\hat{X}}_{\left.k\right|k-1}^{i}\right)\right]}{2}\right) \end{array}$$

(32) q˜ki=qkiqki∑i=1Mqki∑i=1Mqki

During the updating of the state of particles, the particles may be located at the incorrect locations due to the interference of noise. To solve this problem, we fuse the indoor map information to modify the trajectory of the pedestrian. Specifically, we define the particles which are located in the inaccessible areas as the invalid ones, and modify the weight of the *i*-th particle into

(33) $${\hat{q}}_{k}^{i}=\left\{\begin{array}{c} 0,\begin{array}{cc} \mathrm{inaccessible} & \mathrm{areas} \end{array}\\ {\tilde{q}}_{k}^{i},\begin{array}{cc} \mathrm{accessible} & \mathrm{areas} \end{array} \end{array}\right.$$

Finally, we adopt the residual resampling algorithm [34] to optimize the weights of particles further. Based on the optimal particles ${\tilde{X}}_{k}^{i}$ and the corresponding weights ${\hat{q}}_{k}^{i}$, we estimate the locations of the pedestrian by

(34) $${\tilde{X}}_{k}=\sum_{i=1}^{M}{\hat{q}}_{k}^{i}{\tilde{X}}_{k}^{i}$$

## 4. Experimental Results

### 4.1. Environment Layout

**Figure 9:**
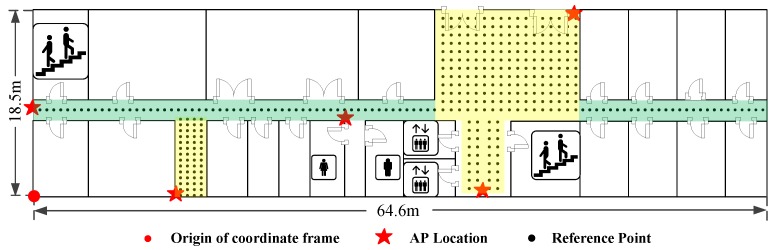
Environment layout.

The experiments are conducted on the same floor with the size of 64.6m×18.5m in a building, as shown in Figure 9. There are five APs and 363 RPs which are labelled with red stars and black dots respectively in the target environment. The distance between every two neighbouring RPs in the green and yellow shaded areas is set as 0.6 m and 0.8 m. The origin of the coordinate frame is labelled with a red dot, while the X- and Y-axis point rightwards and upwards respectively.

**Figure 10:**
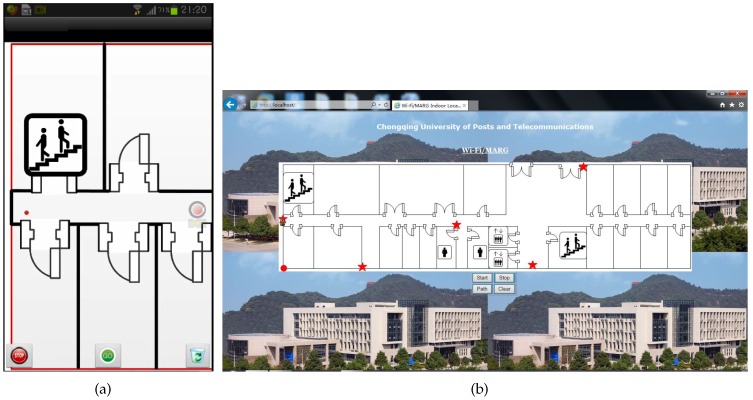
Interface of our developed software. (**a**) Mobile client; (**b**) Remote control.

**Figure 11:**
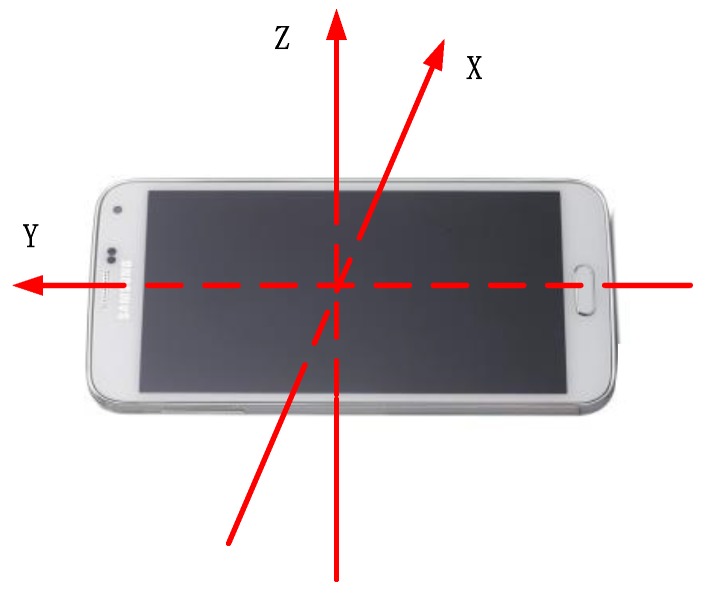
Body coordinate frame with respect to the receiver.

The Galaxy S3 smartphone running under the Android 4.1.2 operation system is selected as the receiver. The interface of our developed software contains two parts, i.e., Android based mobile client (see Figure 10a) and Web page based remote control (see Figure 10b). The body coordinate frame with respect to the receiver is shown in Figure 11. The receiver collects the data from the Wi-Fi module and MARG sensors, and then transmits them to the location server through the Wi-Fi network. The location server estimates the locations of the pedestrian, and then returns the localization results to the receiver and remote control. The refresh rate of the data from the MARG sensors is 50 Hz and the updating rate of the localization is 1 Hz.

### 4.2. Performance of PDR Localization

#### 4.2.1. Stride Length Estimation

To examine the performance of stride length estimation, we invite 10 volunteers with the height ranging from 150 cm to 181 cm for the testing.

● Testing design

Taking the difference of walking speed into account, each volunteer is required to walk along the same path with the length of 100 m under three different walking speeds, namely slow, normal, and fast, to train the stride length model. After the stride length model is obtained, each volunteer is required to walk along another path with the length of 140 m under the random walking speed for the testing.

● Data analysis

The two existing popular methods used to optimize the stride length model are summarized as follows. The first one [35], $M_{1}$, is based on the ratio of the real and estimated walking distances to optimize the calibration coefficient, and then uses Equation (1) to estimate the stride length, $L_{M_{1}}$. The second one [9], $M_{2}$, is based on the height and stride frequency of the pedestrian to construct a linear mathematical model to estimate the stride length, $L_{M_{2}}$. Different from the methods above, the proposed one, $M_{3}$, is based on the BP neural network to optimize the calibration coefficient to estimate the stride length, $L_{M_{3}}$.

**Figure 12:**
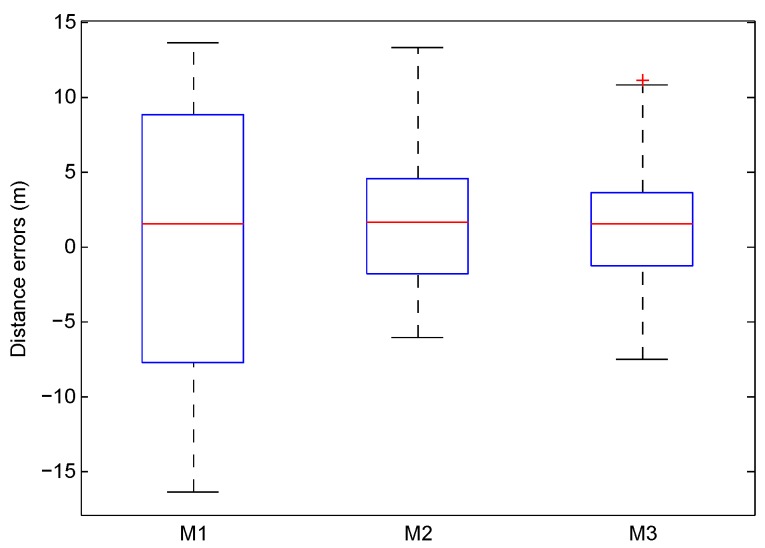
Distance errors between the real and estimated walking distances.

In Figure 12, we use the box-plots to compare the localization errors by using $M_{1}$, $M_{2}$, and $M_{3}$ to estimate the stride length. From this figure, we can find that the interquartile range by $M_{1}$ is larger than the ones by $M_{2}$ and $M_{3}$, which indicates that the localization error by $M_{1}$ is the largest. Based on the upper Whiske, the maximum localization error by $M_{3}$ is the smallest, whereas an abnormal localization error (with a red cross) is resulted by $M_{3}$.

#### 4.2.2. Heading Estimation

**Figure 13:**
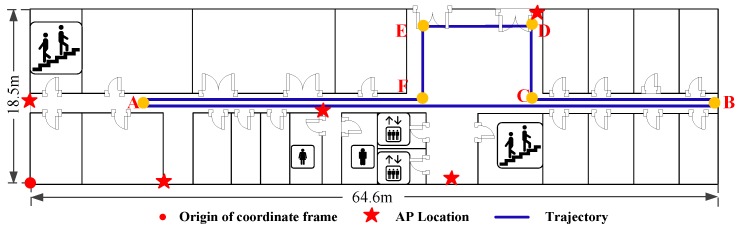
Testbed for heading estimation.

The testbed used for heading estimation is shown in Figure 13. The trajectory of the pedestrian is notated as A→B→C→D→E→F→A with the length of 126.56 m. To investigate the performance of the proposed Complementary Filter Auxiliary EKF (CFAEKF) for heading estimation, we compare it with two existing approaches: the EKF and Gradient Descent Algorithm (GDA) [30].

**Figure 14:**
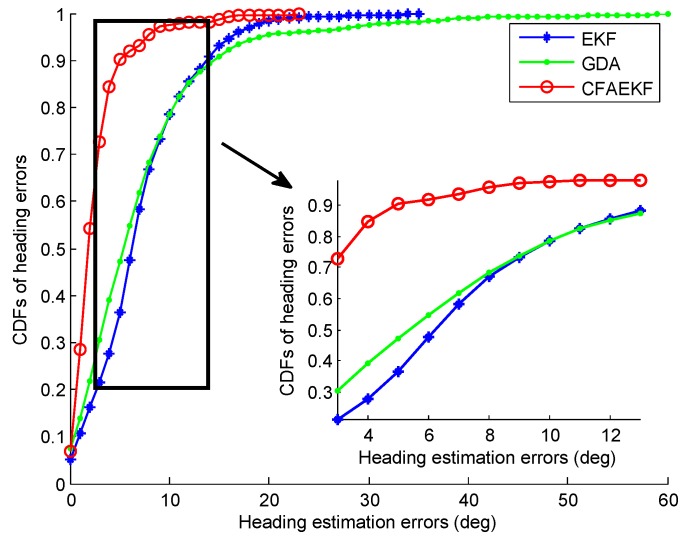
CDFs of errors of heading estimation.

**Figure 15:**
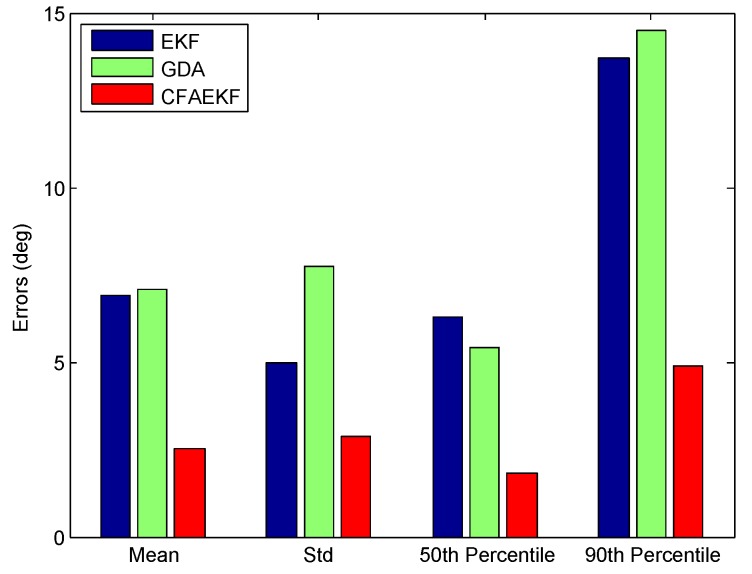
Statistical errors of heading estimation.

**Figure 16:**
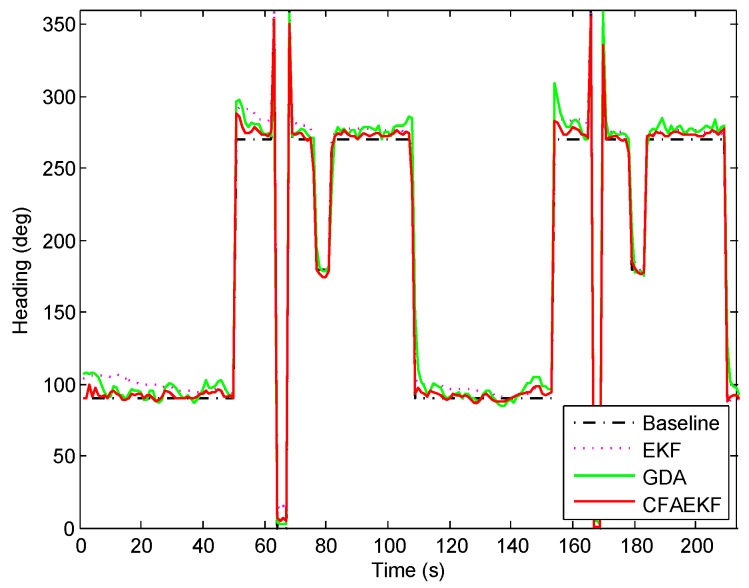
Result of heading estimation.

Figure 14 shows the CDFs of errors of heading estimation by the proposed CFAEKF and the conventional EKF and GDA. From this figure, we can find that the proposed CFAEKF performs best, with most errors of heading estimation falling within the range of $10^{\circ }$, compared with the EKF and GDA. In addition, Figure 15 shows the statistical errors of heading estimation. From this figure, we can find that the 50th and 90th percentile errors by the EKF, GDA, and CFAEKF are $6.3^{\circ }$, $5.4^{\circ }$ and $1.8^{\circ }$, and $13.7^{\circ }$, $14.5^{\circ }$ and $4.9^{\circ }$ respectively; meanwhile, the CFAEKF reduces the mean error by 57.89% and 58.84% from the ones by the EKF and GDA. To illustrate this result clearer, Figure 16 shows the result of heading estimation. From this figure, we can find that the result of heading estimation by the CFAEKF is more similar to the real heading, namely baseline, compared with the ones by the EKF and GDA.

**Figure 17:**
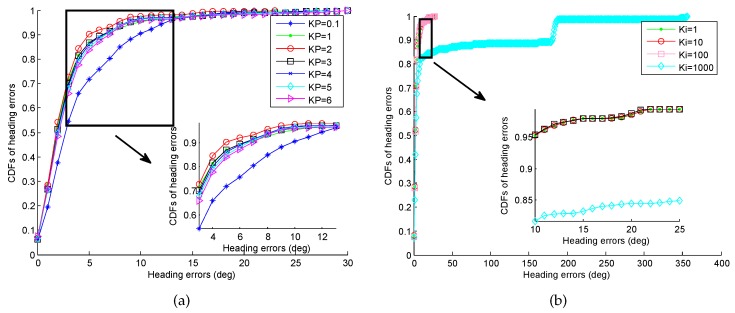
CDFs of errors of heading estimation under different values Kp and Ki. (**a**) Different value Kp; (**b**) Different value Ki.

For the CFAEKF, since the output of the gyroscope is modified by the PI controller, the selection of control parameters has a significant impact on the heading estimation. Figure 17 shows the CDFs of errors of heading estimation under different values Kp and Ki. From these figures, we can find that when the value Kp is larger than 1, the value Kp has a slight impact on the error of heading estimation. When the value Ki is smaller than 100, the impact on the error of heading estimation of value Ki is slight, while as the value Ki increases to 1000, more than 10% of the errors are larger than $150^{\circ }$. Due to the consideration of the sensitivity and stability of our system, we set Kp=2 and Ki=1.

### 4.3. Performance of Wi-Fi Localization

**Figure 18:**
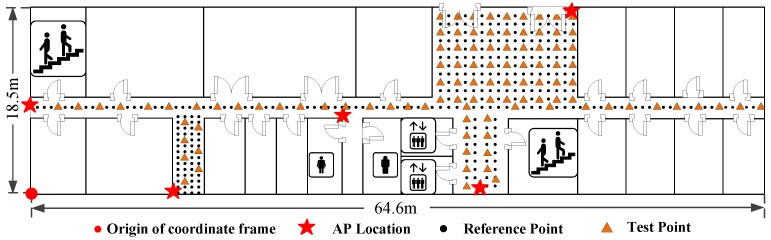
Testbed for static localization.

**Figure 19:**
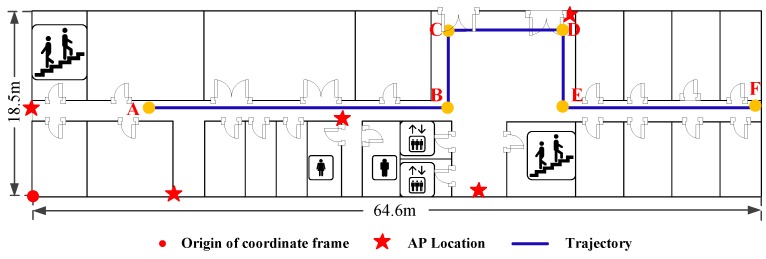
Testbed for dynamic localization.

To investigate the performance of Wi-Fi localization, we design two experiments including the static and dynamic localization. Figure 18 and Figure 19 show the testbeds for the static and dynamic localization respectively. For the static localization, we collect 10 RSS samples at each test point with random orientations of human body, while for the dynamic localization, the pedestrian walks along a given path A→B→C→D→E→F which is labelled by the blue solid line.

● Direction database

The proposed radio map, namely the Direction Database (DD), is constructed from the radio maps with eight different orientations. The Mean Database (MD) is defined as the radio map which is constructed from the mean of the four radio maps with the orientations $0^{\circ }$, $90^{\circ }$, $180^{\circ }$ and $270^{\circ }$. For the comparison, the conventional radio map, namely the Static Database (SD), is constructed by collecting the Wi-Fi RSS at each RP without the blocking of the human body in the fixed orientation.

**Figure 20:**
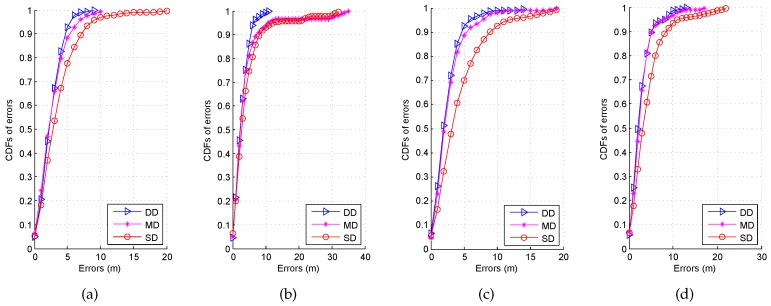
CDFs of errors by using different types of database. (**a**) Orientation 1; (**b**) Orientation 2; (**c**) Orientation 3; (**d**) Orientation 4.

To investigate the impact of different databases on the accuracy of the static localization, we show the CDFs of errors in Figure 20. From this figure, we can find that the localization performance by using the DD is better than the ones by the MD and SD under four different orientations. To illustrate this result clearer, we select five popular performance metrics, i.e., mean error, standard deviation of errors, maximum error, 67% error, and probability of errors over 5 m, to compare the statistical errors by these three types of database in Table 1. Due to the blocking of the human body, the probabilities of errors over 5 m by the MD and SD are higher compared with the DD; meanwhile, the DD reduces the mean error by 30.65% and 36.61% from the ones by the MD and SD under the orientation 2.

● Affinity propagation clustering

Considering both the time overhead and localization accuracy, we propose using the improved affinity propagation clustering for the coarse localization and the inverse matching auxiliary WKNN for the fine localization, namely WKNN + Inverse matching + Improved APC. For the inverse matching method, we search for the K RPs which are geographically close to the one-step prediction location from Equation (26), estimate the RSS at the one-step prediction location by weighting the RSSs at the K selected RPs, and modify the collected RSS at the one-step prediction location by the estimated one.

**Figure 21:**
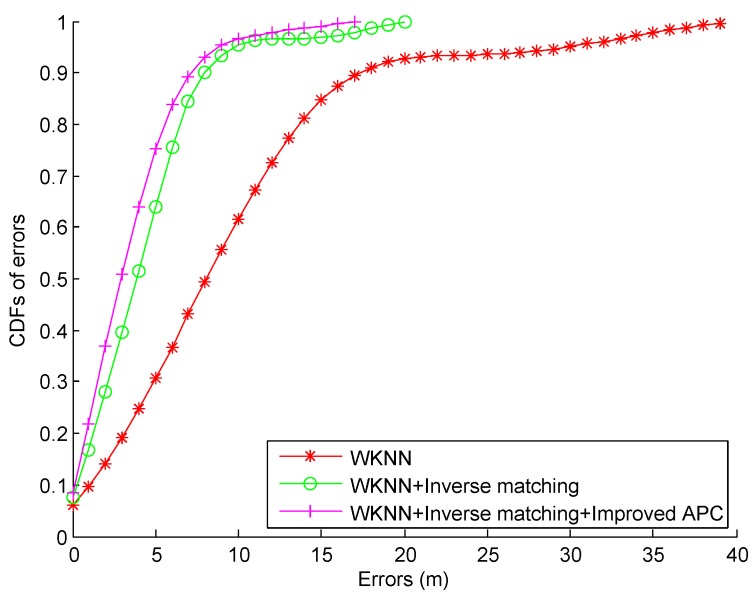
CDFs of errors by using different Wi-Fi localization approaches.

Using the Wi-Fi RSS data collected on the given path (see Figure 19), we compare the CDFs of errors by using the WKNN, WKNN + Inverse matching, and the proposed WKNN + Inverse matching + Improved APC in Figure 21. From this figure, we can find that there is no error over 20 m by the WKNN + Inverse matching and WKNN + Inverse matching + Improved APC, which indicates that the inverse matching approach can effectively decrease the large error probability. In addition, the improved APC is able to reduce the large localization errors further since the process of coarse localization will discard the abnormal RPs which are far away from the corresponding clustering centres. In Table 2, we can find that the proposed approach decreases the mean error by 63.68% and 19.62%, and the 67% error by 61.47% and 20.75% from the ones by the WKNN and WKNN + Inverse matching respectively.

### 4.4. Performance of Fusion System

**Figure 22:**
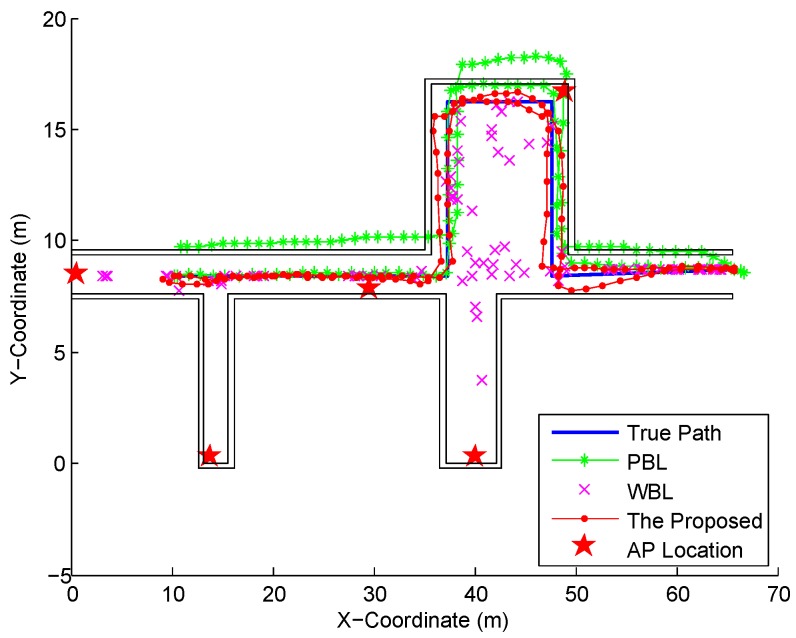
Location tracking by the proposed fusion, PBL, and WBL systems.

**Figure 23:**
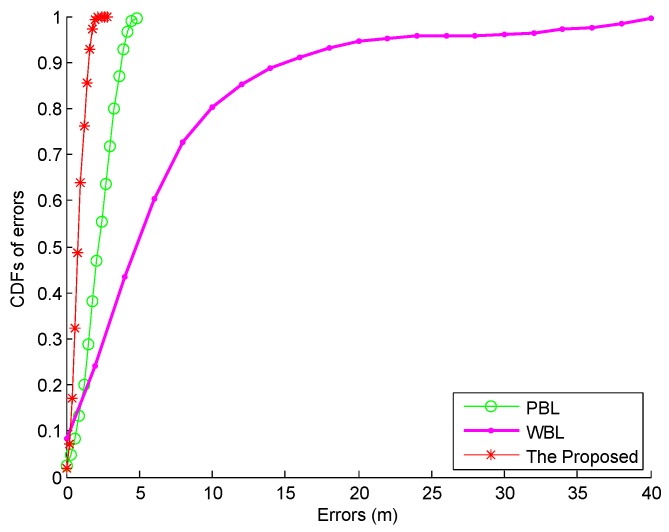
CDFs of errors by the proposed fusion, PBL, and WBL systems.

We compare the location tracking performance of the proposed fusion system and the conventional PDR-based Localization (PBL) and Wi-Fi based Localization (WBL) systems without data fusion in Figure 22. From this figure, we can find that the PBL involves the accumulative error for a long time, while the WBL contains many irregular large errors due to the disturbance of the Wi-Fi signal. As can be seen from Figure 23, the proposed fusion system performs best in localization accuracy due to the fact that it not only avoids the accumulative error by the PBL, but also eliminates the large errors by the WBL. In Table 3, it is found that the mean error by the PBL and WBL are 2.22 m and 6.79 m respectively, which is larger than the one by the proposed fusion system, 0.85 m. In addition, the proposed fusion system decreases the 67% error by 64.41% and 84.85% from the ones by the PBL and WBL respectively.

**Figure 24:**
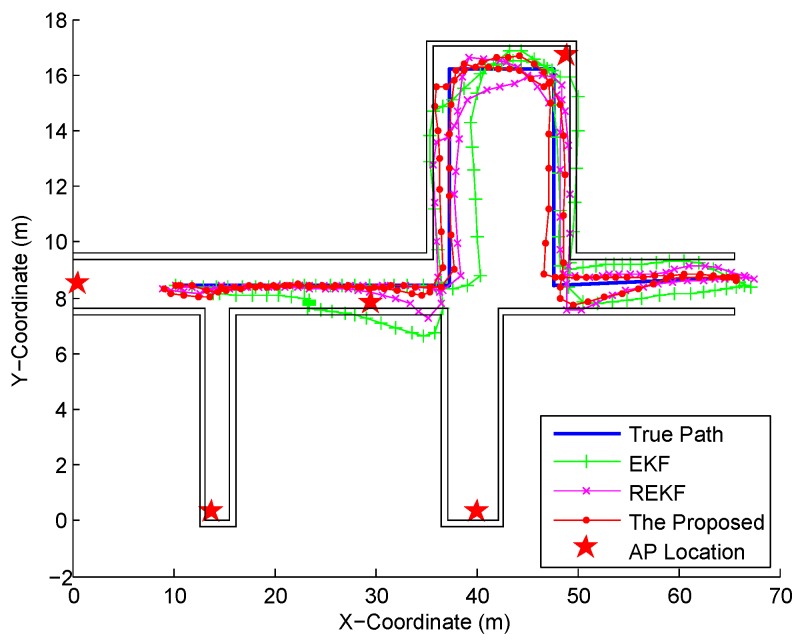
Location tracking by the proposed fusion, EKF, and REKF systems.

**Figure 25:**
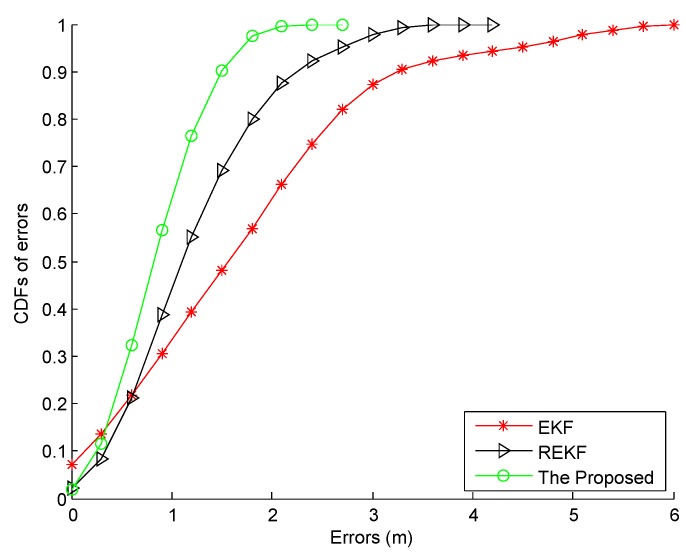
CDFs of errors by using the proposed fusion, EKF, and REKF systems.

We continue to compare the location tracking performance of the proposed fusion system and the conventional fusion systems, EKF and Robust EKF (REKF) in Figure 24. Compared with the EKF and REKF, the proposed fusion system achieves the best location tracking performance, especially when the pedestrian makes a turn. The CDFs of errors by using different fusion systems are shown in Figure 25. Obviously, the proposed fusion system performs better in localization accuracy than the EKF and REKF since its particle updating scheme results in the well data fusion by combining the optimal IDF and map information. As illustrated in Table 4, the proposed fusion system decreases the mean error by 49.40% and 29.75% and standard deviation of errors by 63.64% and 38.03% from the ones by the EKF and REKF respectively.

**Figure 26:**
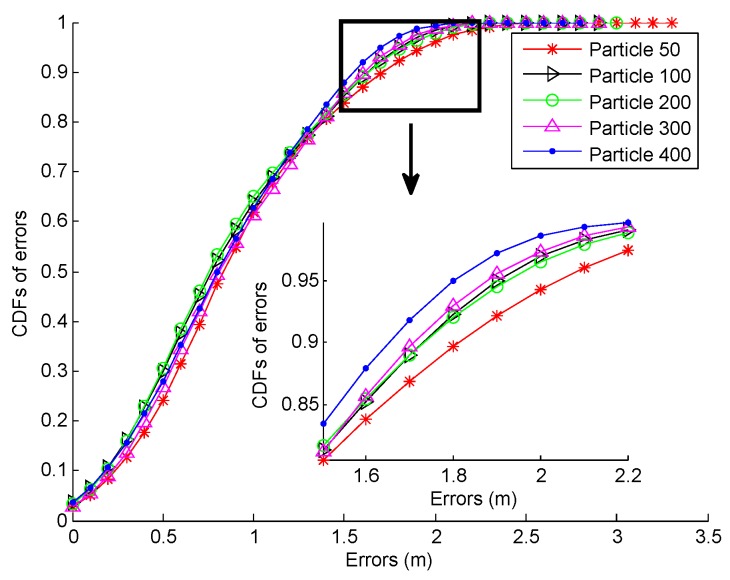
CDFs of errors under different numbers of particles.

**Figure 27:**
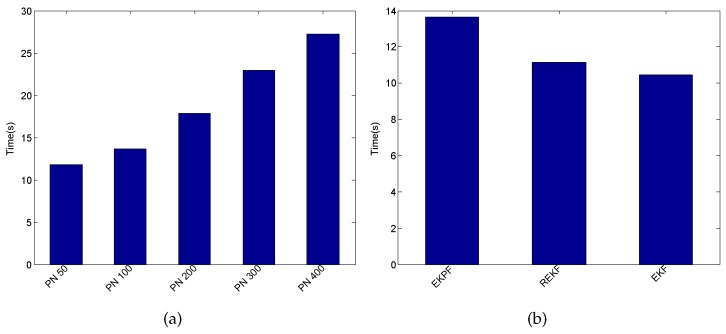
Comparison of computation time. (**a**) Under different number of particles; (**b**) Under different fusion systems.

Finally, we investigate the computation time required by the proposed fusion system. The target user walks along a given path A→B→C→D→E→F→E→D→C→B→A with the length of about 142 m, as shown in Figure 19. This process takes this user about two minutes. After that, we calculate the computation time in total involved in the localization process by the proposed system under different particle numbers. In our experiment, all the calculations are conducted on a desktop with the Intel (R) Core (TM) i3-3240 CPU and 4 GB RAM. As can be seen from Figure 27a, the computation time gradually increases with the increase in particle number, whereas the particle number has a slight impact on localization accuracy according to the CDFs of errors in Figure 26. The computation time required by the three different fusion systems mentioned above is shown in Figure 27b. Based on the results in Figure 25 and Figure 27b, we can find that although the proposed fusion system consumes a little more computation time compared with the EKF and REKF, its related localization accuracy is much higher than the other two.

## 5. Conclusions

In this paper, we propose a new Wi-Fi/MARG indoor localization system by fusing the data from the Wi-Fi module and MARG sensors, and then integrating the indoor map information to improve the localization accuracy. For the PDR, we employ the complementary filter auxiliary EKF to estimate the heading and the velocity of the pedestrian by using the BP neural network, while for the Wi-Fi localization, we rely on the improved affinity propagation clustering approach to improve the localization accuracy of the WKNN. The proposed integration system can avoid the accumulative error of the PDR, as well as reducing the large error probability of the Wi-Fi localization which is caused by the disturbance of the Wi-Fi signal. Furthermore, we rely on our developed software, including the Android based mobile client, Web page for remote control, and location server, to verity the effectiveness of the proposed system in the real indoor environment. In the future, we will focus on the design of an effective and efficient radio map construction approach to reduce the time and labor cost involved in the site survey of the large-scale indoor environment.

## Figures and Tables

**Table 1:** Statistical errors by using different types of database.

Performance Metrics	Types of Database	Orientation 1	Orientation 2	Orientation 3	Orientation 4
	SD	3.43	4.21	4.15	4.06
Mean error (m)	MD	2.54	3.85	2.66	2.71
	DD	2.40	2.67	2.31	2.54
	SD	3.02	5.52	3.76	3.94
Standard deviation of errors (m)	MD	1.99	5.83	2.60	2.57
	DD	1.51	1.97	2.02	2.22
	SD	19.57	31.81	18.63	21.78
Maximum error (m)	MD	9.76	34.13	18.06	16.65
	DD	8.21	10.29	13.67	12.04
	SD	4.70	4.60	4.70	4.60
67% error (m)	MD	2.90	3.10	2.90	3.10
	DD	2.70	2.90	2.70	3.10
	SD	19	22	24	24
Probability of errors over 5 m (%)	MD	8	18	10	7
	DD	3	12	6	8

**Table 2:** Statistical errors by using different Wi-Fi localization approaches.

Performance Metrics	WKNN	WKNN + Inverse Matching	WKNN + Inverse Matching + Improved APC
Mean error (m)	9.41	4.25	3.41
Standard deviation of errors (m)	7.83	3.49	2.90
Maximum error (m)	38.95	19.01	16.22
67% error (m)	10.90	5.30	4.20

**Table 3:** Statistical errors of the proposed fusion, PBL, and WBL systems.

Performance Metrics	The Proposed Fusion	PBL	WBL
Mean error (m)	0.85	2.22	6.79
Standard deviation of errors (m)	0.44	1.05	7.69
67% error (m)	1.05	2.95	6.93
90% error (m)	1.56	3.81	15.10

**Table 4:** Statistical errors of the proposed fusion, REKF, and EKF systems.

Performance Metrics	The Proposed Fusion	REKF	EKF
Mean error (m)	0.85	1.21	1.68
Standard deviation of errors (m)	0.44	0.71	1.21
67% error (m)	1.05	1.48	2.25
90% error (m)	1.56	2.25	3.28
